# High dose teriparatide (rPTH_1-34_) therapy increases callus volume and enhances radiographic healing at 8-weeks in a massive canine femoral allograft model

**DOI:** 10.1371/journal.pone.0185446

**Published:** 2017-10-11

**Authors:** Kohei Nishitani, Zachary Mietus, Christopher A. Beck, Hiromu Ito, Shuichi Matsuda, Hani A. Awad, Nicole Ehrhart, Edward M. Schwarz

**Affiliations:** 1 Center for Musculoskeletal Research, University of Rochester Medical Center, Rochester, NY, United States of America; 2 Department of Orthopaedics Surgery, Graduate School of Medicine, Kyoto University, Kyoto, Japan; 3 Department of Biomedical Engineering, University of Rochester, Rochester, NY, United States of America; 4 Department of Orthopedics, University of Rochester Medical Center, Rochester, NY, United States of America; 5 Department of Biostatistics and Computational Biology, University of Rochester Medical Center, Rochester, NY, United States of America; 6 Department of Clinical Sciences, Colorado State University, Fort Collins, CO, United States of America; Nanjing Medical University, CHINA

## Abstract

Small animal studies have demonstrated significant high-dose recombinant parathyroid hormone_1-34_ (rPTH_1-34_) effects on intercalary allograft healing. Towards a human adjuvant therapy to decrease non-unions, we evaluated rPTH_1-34_ safety and efficacy in a clinically relevant canine femoral allograft model. Adult female mongrel hounds (n = 20) received a 5cm mid-diaphyseal osteotomy reconstructed with a plated allograft, and were randomized to: 1) Placebo (n = 5; daily saline), 2) Continuous rPTH_1-34_ (n = 7; 5 μg/kg/day s.c. from day 1–55 post-op), or 3) Delayed rPTH_1-34_ (n = 8; 5 μg/kg/day s.c. from day 14–28 post-op). Safety was assessed by physical behavior and blood calcium monitoring. Cone beam CT (CB-CT) was performed on days 14, 28 and 56 post-op to assess 2D cortical healing, 3D bone volume, and Union Ratio. Biomechanical testing and dynamic histomorphometry were also performed. The high drug dose was poorly tolerated, as most dogs receiving rPTH_1-34_ had to be given intravenous saline, and one dog died from hypercalcemia. Continuous rPTH_1-34_ significantly increased 2D healing and callus volumes at 4-weeks versus Placebo, and sustained the significant increase in cortical union at 8-week (p<0.05). These rPTH_1-34_ effects were confirmed by histomorphometry, revealing significant increases in mineral apposition rates (MAR) on host bone and graft-host junctions (p<0.05). Delayed rPTH_1-34_ significantly increased callus volume and MAR at 8 weeks (p<0.05). Although no biomechanical differences were observed, as expected for early healing, the results demonstrated that 2D RUST scoring significantly correlated with torsional biomechanics (p<0.01). In conclusion, 8-weeks of intermittent high-dose rPTH_1-34_ treatment significantly increases callus formation and accelerates bony union of intercalary massive allografts in a clinically relevant canine model, but with serious side-effects from hypercalcemia.

## Introduction

The clinical management of critical (>3cm) segmental defects remains challenging with poor outcomes that worsen over time [1, 2]. Despite surgical advances, transplantation of massive allografts remains a general orthopaedic procedure due to graft availability, and donor site morbidity issues with the other approaches [3, 4]. Thus, the quest for a practical adjuvant therapy remains a high priority. One possibility is teriparatide (recombinant parathyroid hormone_1-34_ (rPTH_1-34_), which is FDA-approved for the treatment of osteoporosis) [5], which has been investigated in normal facture healing, delayed unions and non-unions [6–8]. However, a recent systematic review of these clinical studies concluded that evidence of efficacy remains anecdotal [9]. In contrast, many groups have demonstrated significant rPTH_1-34_ effects on bone healing in various small animal models [10–16], with high doses (~100x the FDA approved 20 μg (~0.3μg/kg/day) subcutaneous dosing regimen for osteoporosis therapy). Interestingly, we failed to see efficacy with lower doses, and observed that only 2-weeks (days 14–28 post-op) of high dose therapy was necessary to see significant healing in a murine femoral allograft model [17]. High doses (5 μg/kg/day) were also needed for efficacy in canine models of implant fixation, bone grafting, and prosthesis osteointegration [18–20]. Thus, given that canines are an ideal large animal model of massive allografting due to the clinical significance of veterinary care for osteosarcoma, and this species is known to be particularly sensitive to PTH-induced hypercalcemia [21], formal safety and efficacy studies of high dose rPTH_1-34_ are warranted.

Another major challenge is the lack of a clinically relevant, translational, and objective outcome measure to prove massive allograft healing, which in animals is primarily assessed by ex vivo torsion testing. Since this cannot be performed on humans, most clinical studies use subjective semi-quantitative radiology (union of 3 or 4 cortices from x-rays) as their primary outcome measure [22, 23]. To improve this, we developed the 3D-CT based Union Ratio, which quantifies connectivity between the host and allograft bone, and correlates with the torsional biomechanics of murine femoral allografts [24, 25]. We also demonstrated the utility of the Union Ratio to assess biomechanical healing of canine femoral allografts, and corroborate the results with dynamic histomorphometry [26, 27]. Based on these advances, we hypothesized that high dose intermittent rPTH_1-34_ therapy following massive allografting: 1) is well tolerated, 2) significantly increases callus volume and accelerates host-allograft healing, and 3) only needs to be given from 2 to 4 weeks post-op to achieve maximum efficacy. Thus, the purpose of this study was to evaluate the effects of continuous rPTH_1-34_ therapy (every day follow surgery), and delayed-short course rPTH_1-34_ therapy (daily from 2-weeks to 4-weeks after surgery), versus placebo, on radiographic, biomechanical and histological healing of a 5cm mid-diaphyseal intercalary allograft over 8-weeks, in a canine femur critical defect model.

## Materials and methods

This study was carried out in strict accordance with the recommendations in the Guide for the Care and Use of Laboratory Animals of the National Institutes of Health. The protocol (UCAR 2012–027) was approved by the University Committee on Animal Resources of the University of Rochester, Rochester, NY. All surgery was performed under general anesthesia, and all efforts were made to minimize suffering. The canines were housed as a group before surgery under the consideration of the sociality of the animal, but housed separately after surgery to avoid excessive stress on the affected limb. Skeletally mature mongrel hounds (n = 20, 23.0 ± 1.5 Kg) were purchased from Marshall BioResources (Hamilton, NY, USA), and their general good health was confirmed by veterinarians from physical exams and x-rays. Dogs were randomized into the 3 treatment Groups based on weight. Group 1: Placebo (n = 5) daily subcutaneous (saline, Group 2: Continuous rPTH_1-34_ (n = 7; 5 μg/kg/day teriparatide; gift of Lilly Inc., Indianapolis, IN, USA) from day 1 post-op to sacrifice. Group 3: Delayed rPTH_1-34_ (n = 8; 5 μg/kg/day) from day 14 to 28 post-op. This experiment was performed in three independent periods during 3 years. Because of the exclusions in the first and second periods which would be described later, the inequality among groups remained.

### Surgery and in vivo procedures

All surgery was performed by N.E. and K.N. in 3 cohorts, as previously described [27]. Under a general anesthesia, a lateral incision was made on the right thigh from the level of the proximal end of the greater trochanter to the proximal end of the lateral condyle. The superficial leaf of the fascia lata muscle was incised and thebiceps femoris muscle was retracted laterally to reveal the femur. A 5 cm femoral segment was removed at the midshaft of the femur diaphysis using cold saline irrigation and reconstructed with a fresh frozen cortical allograft (Veterinary Transplant Services, Inc, Kent, WA, USA), which was stabilized using a 12-hole titanium 3.5 DCP plate and screws (gift of Synthes Inc. West Chester, PA) by using the AO compression technique. Antibiotics (cefazolin) were given prior to the skin incision and every 2 hours during surgery. Post-operative analgesia was achieved with a fentanyl patch placed before surgery, and oral morphine (1–2 mg/kg orally) delivered every 8 hours as needed. Recovery was monitored every 12 hours for one week using a semiquantitative scoring system, which based on the pain and activities [27]. Briefly, the recovery score consists of lameness (0: not detectable– 6: no ambulatory), swelling (0: none– 3: sever), pain on palpation (0: none– 4: dog will not allow examination) and pain on joint motion (0: none– 4: dog will not allow examination). Blood calcium was measured 1–2 hrs after rPTH_1-34_ injection, and dogs with hypercalcemia (serum ionized calcium level 1.60 mmol/L), or dehydration symptoms perceived to be drug-related, received i.v. saline infusions. In vivo cone beam CT (CB-CT) scans were taken on days 14, 28 under general anesthesia [27]. For dynamic histomorphometry, all dogs received alizarin red (25 mg/kg iv) and calcein green (25 mg/kg iv) 8 days and 1 day before sacrifice on day 56 post-op respectively [27]. The dogs were euthanized via sodium pentobarbital overdose on day 56, and bilateral femurs were harvested for ex vivo CB-CT scan, biomechanical testing, and undecalcified histomorphometry.

### Radiographic analysis

3D reconstruction of the primary DICOM files from the CB-CT scans was performed using Amira 5.4 (Visage Imaging, Inc, Andover, MA) to quantify volume of the: host femur, allograft, mineralized callus, and the femoral plate (internal control) [27]. For normalization, two blinded operators quantified all volumes independently (ICC = 0.89), and average values were used for statistics. 2D reconstruction was performed using Amira, and sagittal and coronal images at the center of the femur were analyzed by a modified Radiographic Union Scale for Tibial fractures (RUST)[22], and a modified Radiographic Union Score for Hip (RUSH) [23]. Scores were independently determined by two blinded board certificated orthopaedic surgeons, and the average was used for statistics. The Union Ratio was determined with Matlab (The Mathworks, Natick, MA) by a single observer blinded to the treatments [27].

### Biomechanical test

Destructive torsion testing was performed on bilateral femurs using an Instron Electropulse 10,000 (Instron Corp., Canton, MA) [27]. Disarticulated femurs were cemented into potting boxes using polymethylmeth- acrylate (PMMA) in a custom jig. A compressive preload of 20 N was applied to the constructs to establish a reference configuration, and the proximal femur was externally rotated at 10°/second (0.17 radians/second) until failure to quantify: yield torque, ultimate torque, torsion rigidity and work to ultimate torque.

### Histomorphometry

Following biomechanical testing, the allografted femurs were processed for undecalcified histomorphometry by a commercial vendor (Alizée Pathology, LLC, Thurmont, MD), who produced representative Sanderson’s rapid bone stain (Surgipath, Richmond, IL, USA) counterstained with acid fuchsin, and quantified the mineral apposition rate (MAR) within eight regions of interest (ROI) per slide via automated histomorphometry of fluorescent images (S1 Fig).

### Statistics

Intraclass correlation coefficient (ICC) was used to determine concordance of 3D volumetric analysis and radiographic scoring. Two–way repeated measures ANOVA with Turkey post hoc test was used to compare 3D callus volume and radiographic scores. One-way ANOVA with Turkey post hoc test was used for Union Ratio and biomechanical analyses. Pearson correlation coefficient was determined to correlate quantitative radiology and biomechanics. Correlations between radiographic score and biomechanics were assessed by Spearman coefficient. For all tests p<0.05 was considered significant.

## Source of funding

National Institutes of Health, Grant numbers: P30 AR069655, P50 AR054041, R01 DE019902 and R01 AR070613

## Results

**Fig 1:**
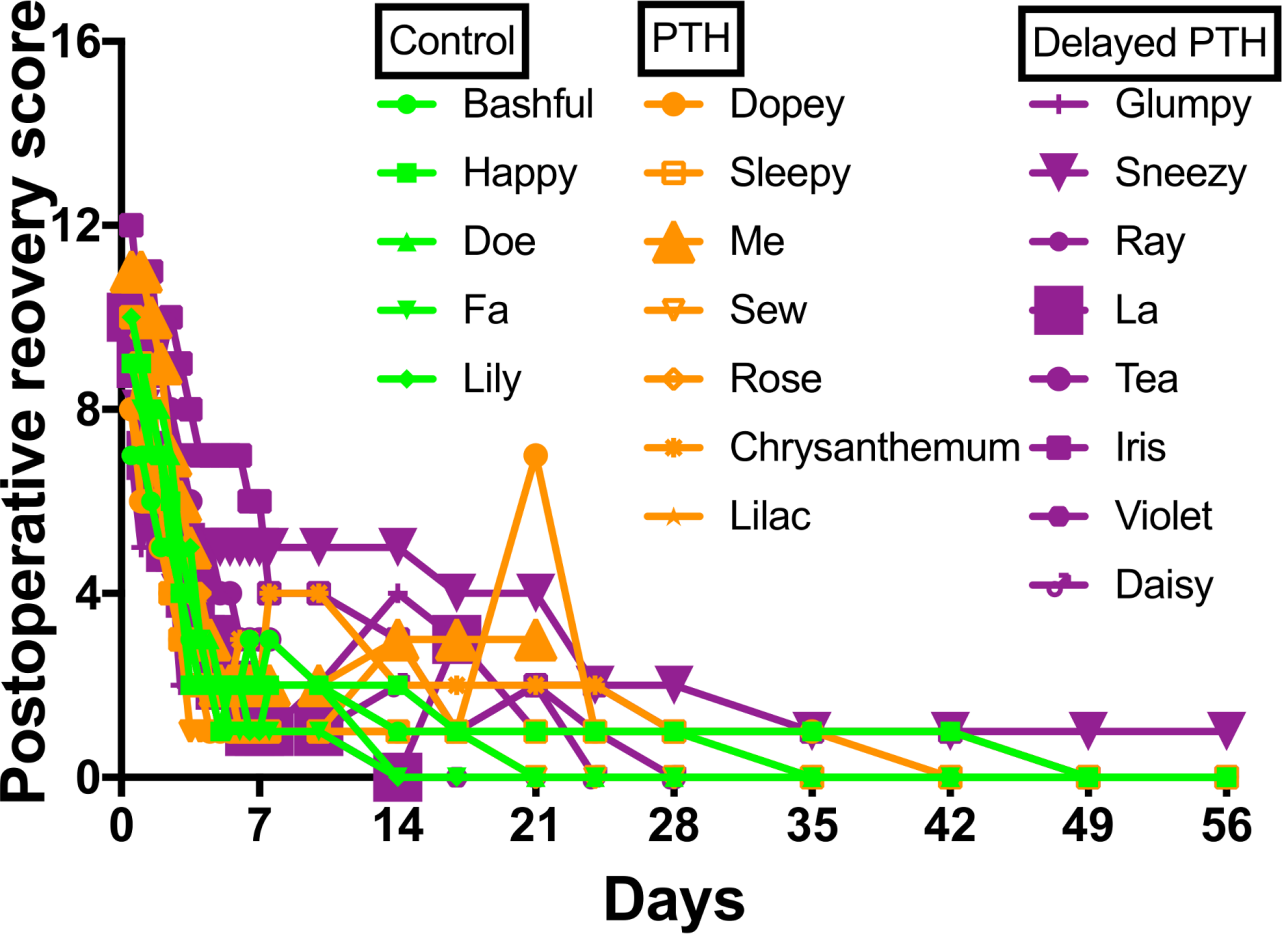
Postoperative recovery following femoral massive allograft surgery. Postoperative recovery scores were obtained on all dogs as described in Materials and Methods, and the total score for each animal over time is shown. No differences between Group 1 (Control) shown in green lines and symbols, Group 2 (PTH) shown in orange lines and symbols, and Group 3 (Delayed PTH) shown in violet lines and symbols; or Cohort 1 (canine subjects: Bashful, Happy, Dopey, Sleepy, Grumpy & Sneezy), Cohort 2 (canine subjects: Doe, Fa, Me, Sew, Ray, La & Tea) and Cohort 3 (canine subjects: Lily, Rose Chrysanthemum, Lilac, Iris, Violet & Daisy); were observed. All dogs discontinued their postoperative analgesia by day 7. Of note are the discontinued lines for La and Me, who died on days 19 and 22 from complications associated with hypercalcemia and *Pseudomonas aeruginosa* infection respectively. Also of note is Sneezy’s continuously high recovery score due to habitual patellar dislocation. These three dogs were excluded from further evaluation for these reasons.

The massive allograft surgery was well tolerated by all dogs included in the study (Fig 1). No complications, including hypercalcemia, were observed in any of the dogs in Group 1. In contrast, 10 out of 15 dogs that received rPTH_1-34_ suffered from hypercalcemia or drug-related dehydration, and were treated with saline transfusions. This was unexpected based on prior canine and human studies that used this dosing regimen [18–20, 28]. Equally surprising were the highly variable side effects, as 1 dog in Group 2, and 4 dogs in Group 3, received all scheduled rPTH_1-34_ injections without interventions. In the most serious case, a dog died with multiple pathologies from the hypercalcemia after only 3 rPTH_1-34_ injections (S2 Fig). One dog from Group 2 died and excluded from sepsis because of the *Pseudomonas aeruginosa* infection. There was one more exclusion because of the habitual patella dislocation from Group 3. Based on these complications, additional dogs were added to Groups 2 and 3 in the third cohort to ensure completion of the study with n ≥ 5, such that 17 dogs completed the study (n = 5 in Group 1; n = 6 in Group 2; and n = 6 in Group 3).

**Fig 2:**
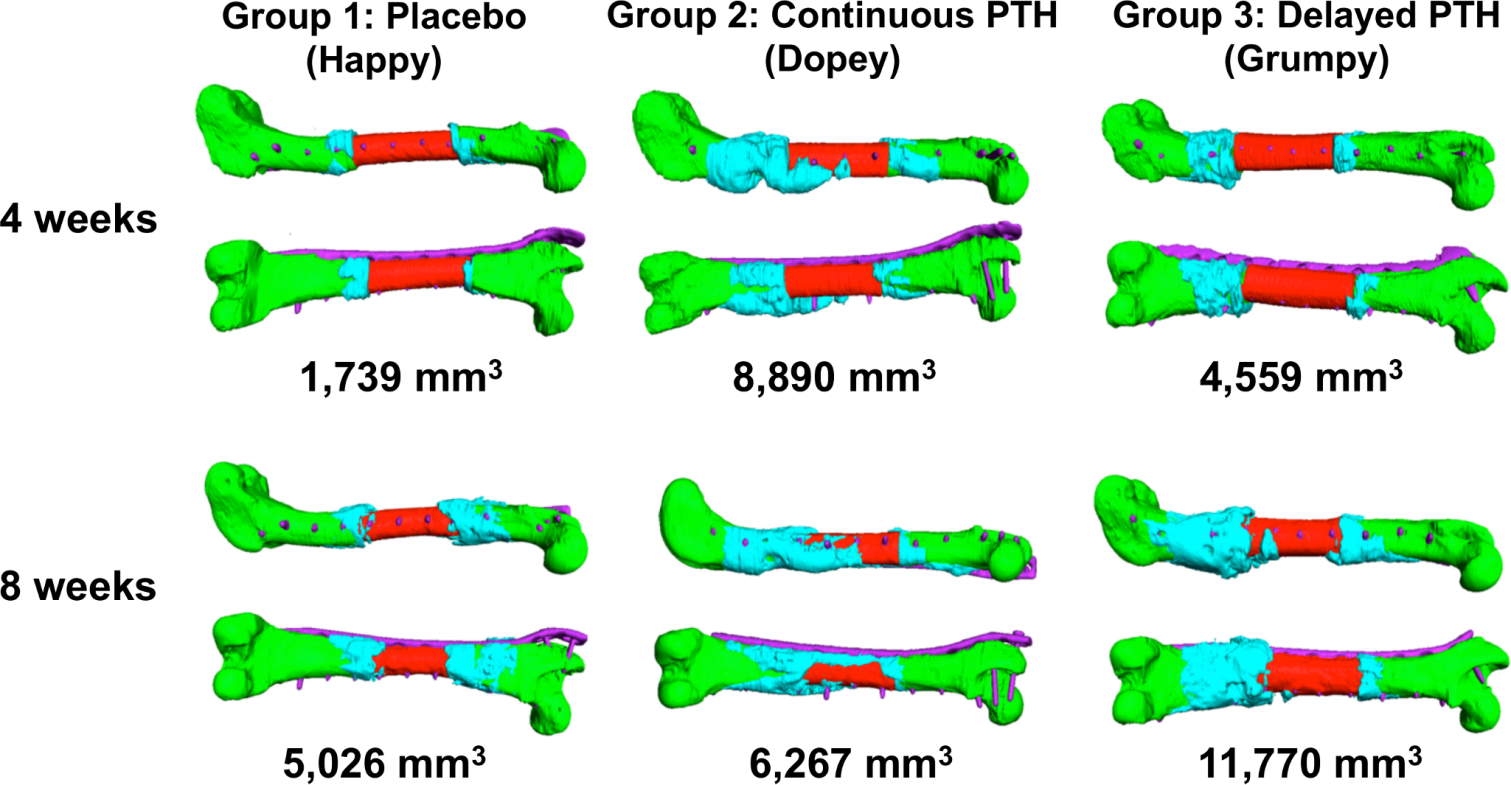
Volumetric CB-CT assessment of callus bone volume. Volumetric rendering of the CB-CT data was performed as described in Materials and Methods, and representative 3D reconstructed images of the plate (purple), host bone (green), allograft (red) and calcified callus (turquoise) of the median dog in each group are shown at 4 and 8-weeks post-op. Of note are: the minimal callus that forms at the graft-host junction in Placebo, the robust new bone formation at 4-weeks that remodels to bridging callus from Continuous PTH at 8-weeks, and the large unremodeled callus at the graft-host junction for Delayed PTH.

CB-CT revealed no callus formation at 2-weeks post-op, while 3D reconstruction of the images at 4-weeks demonstrated initial callus formation in Group 1, robust callus formation in Group 2, and moderate callus formation in Group 3 (Fig 2). By 8-weeks mature callus was restricted to the graft-host junctions in the placebo treated dogs. Remarkably, the large callus in Group 2 was remodeled, and spanned from the proximal to the distal ends of the host bone over some sections of the allograft. Interestingly, the callus surrounding the graft-host junctions remained enlarged in Group 3 at 8-weeks. In the most of animals in all three groups, larger callus tended to be formed around the distal fracture gap than the proximal gap. Quantification of the callus volume confirmed the significant 2.6-fold increase in Group 2 at 4-weeks, and 1.9-fold increase in Group 3 at 8-weeks, versus placebo (Table 1). However, no differences in Union Ratio were observed (S3 Fig).

**Table 1:** Differential effects of continuous and delayed PTH treatment on callus volume during massive femoral allograft healing.

	2 weeks	4 weeks	8 weeks
Group 1: Control	0.00 ± 0.00	1854 ± 1386	4305 ± 1856
Group 2: PTH	0.00 ± 0.00	4849 ± 2638 *	6985 ± 2791
Group 3: Delayed PTH	0.00 ± 0.00	4689 ± 2055	8124 ± 2350 **

Callus volumes were determined from the 3D rendered CB-CT scans as described in Materials and Methods, and the data are presented as the mean +/- SD.

**: p < 0.01

*: p < 0.05 in 2-way repeated measures ANOVA with Turkey post hoc test

**Fig 3:**
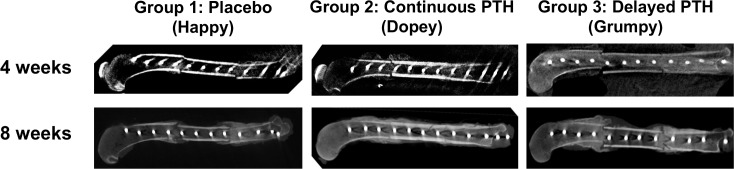
2D CB-CT assessment of femoral intercalary allograft healing. 2D sagittal images from the center of the allografted femurs described in Fig 2 are shown to illustrate cortical bone healing in representative dogs from the 3 groups at 4 and 8-weeks post-op. Of note are: the limited callus and bridging cortical bone in Placebo, the remodeled callus and cortical union in Continuous PTH, and the remodeled callus highlighting fracture gaps in Delayed PTH.

**Fig 4:**
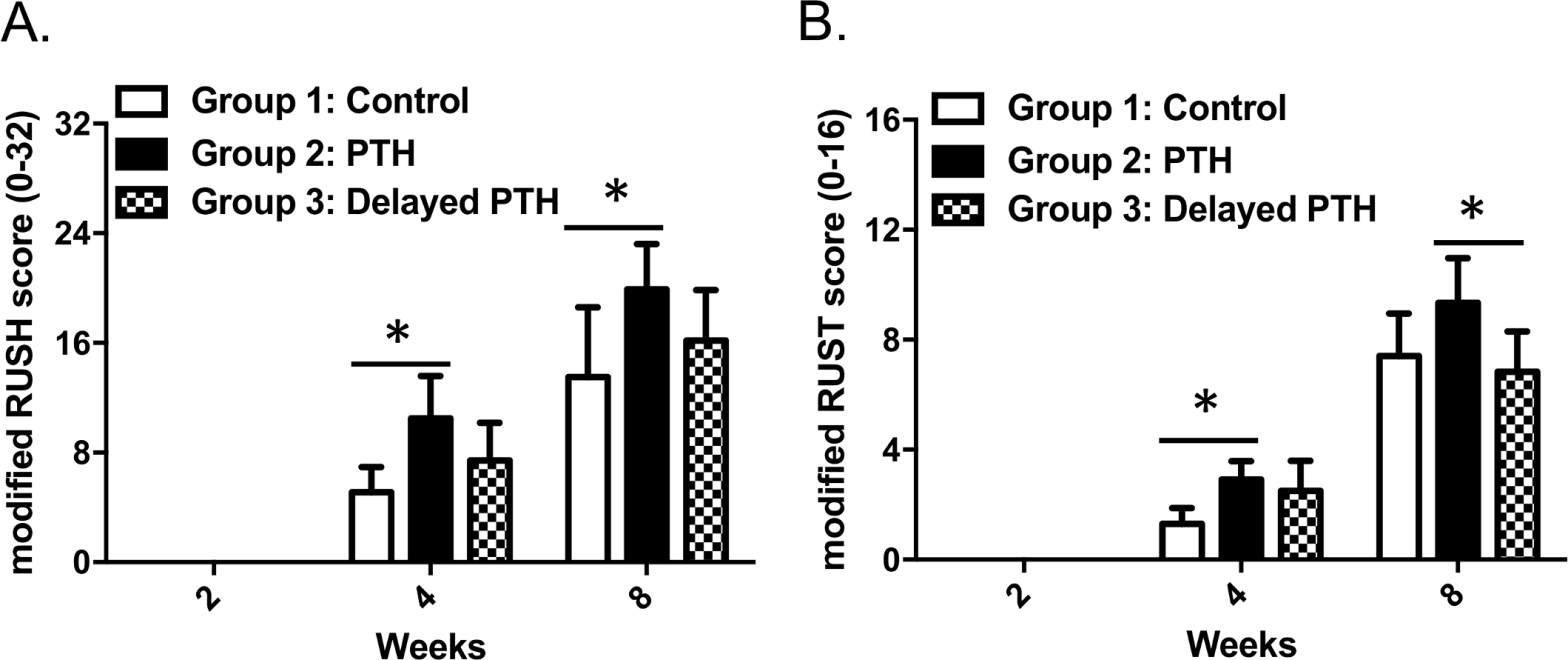
Continuous PTH therapy accelerates cortical union during intercalary allograft healing. Two radiographic healing scores ((A) modified RUSH score and (B) modified RUST) was used to quantify 2D radiographic healing of the fracture gaps at the proximal and distal graft-host junctions of allografted femurs at 2, 4 and 8-weeks. Two board certificated orthopaedic surgeons independently scored each allografted femur at each time point blinded to the treatment, and the average was used as the score for each data point. The ICC for the modified RUSH scores = 0.74. The ICC for the modified RUST scores = 0.83. The data are shown as mean ± SD. *: p < 0.05 in 2-way repeated measures ANOVA with Turkey post hoc test.

The 2D reconstructed images confirmed the accelerated healing of fracture gaps in rPTH_1-34_ treated dogs (Fig 3). Callus formation was observed at 4-weeks in Groups 2 and 3, mainly on the distal side of the distal host-allograft junction, however almost no callus bridge was observed at this time. Evidence of rPTH_1-34_ accelerated healing was also observed at 8 weeks, as dogs in Group 3 displayed a large callus in both proximal and distal fracture gaps, although fracture lines were clearly evident. Greater healing was observed in Group 2, as the callus appeared to be more calcified and decreased in size from remodeling. Moreover, evidence of bridging fracture callus was present on all 4 cortices, although fracture lines were still visible in some cortexes. These findings were confirmed by semiquantitative scoring (Fig 4). Of note is that only dogs in Group 2 had RUSH scores >16, and RUST scores >8, indicating that the fracture lines disappeared in a cortex. Interestingly, despite the large callus size, Group 3 dogs did not show any improvement in their radiographic score, mainly because of the remaining fracture gaps at the graft-host junctions.

Biomechanical testing failed to demonstrate any significant differences between the 3 Groups (Table 2). Interestingly, the values for all parameters in Group 2 were greater, and 3 of for parameters in Group 3 were less than placebo, but also displayed a large degree of variability. However, linear regression analyses showed that RUST scoring achieved moderate correlation to the Yield Torque (R = 0.63), Ultimate Torque (R = 0.77), Torsion rigidity (R = 0.69) and Work to TUlt (R = 0.60) (Table 3). RUSH scoring only showed weak correlation to the Ultimate Torque (R = 0.50) (Table 3). There was no correlation between callus size or Union Ratio with any biomechanical properties.

**Table 2:** PTH treatment does not affect biomechanical healing of intercalary allografts at 8-weeks.

	Yield Torque	Ultimate Torque	Torsion rigidity	Work to TUlt
	[N.m]	[N.m]	[N.m/(rad/m)]	[(N.m)*(rad/m)]
Contralateral side of the Group 1 (normal femur)	22.3 ± 2.71	34.6 ± 3.70	11.2 ± 0.63	81.3 ± 13.0
Group 1: Control	3.62 ± 3.08	6.24 ± 6.36	3.45 ± 3.42	8.29 ± 7.86
Group 2: PTH	4.20 ± 3.61	7.20 ± 6.10	3.60 ± 2.23	12.94 ± 10.17
Group 3: Delayed PTH	2.76 ± 0.96	4.08 ± 1.32	1.69 ± 0.91	10.89 ± 10.22

Canine of normal femurs and allografted femurs harvested at 8-weeks were torsion tested to failure as described in Materials and Methods, and the data are presented as the mean +/- SD. No significant differences between Groups were observed.

**Table 3:** Correlations between radiographic and biomechanical outcomes of intercalary femoral allograft healing at 8-weeks.

Correlations between Radiographic and Biomechanical test	Yield Torque	Ultimate Torque	Torsion rigidity	Work to TUlt
	[N.m]	[N.m]	[N.m/(rad/m)]	[(N.m)*(rad/m)]
Callus Volume	0.06	0.01	-0.11	0.20
Union Ratio	-0.02	0.02	0.04	-0.06
RUSH	0.31	0.50 *	0.33	0.36
RUST	0.63 **	0.77 ***	0.69 **	0.60 **

The 8-week data from all 17 dogs in the study was combined to assess correlations between the radiographic and biomechanical outcome measures. The Pearson r value is reported for 3D quantitative outcomes (Callus Volume and Union Ratio) versus biomechanical outcomes, and Spearman rho is reported for 2D radiology scores (RUSH and RUST) versus biomechanical outcomes.

***: p < 0.001

**: p < 0.01

*: p < 0.05.

**Fig 5:**
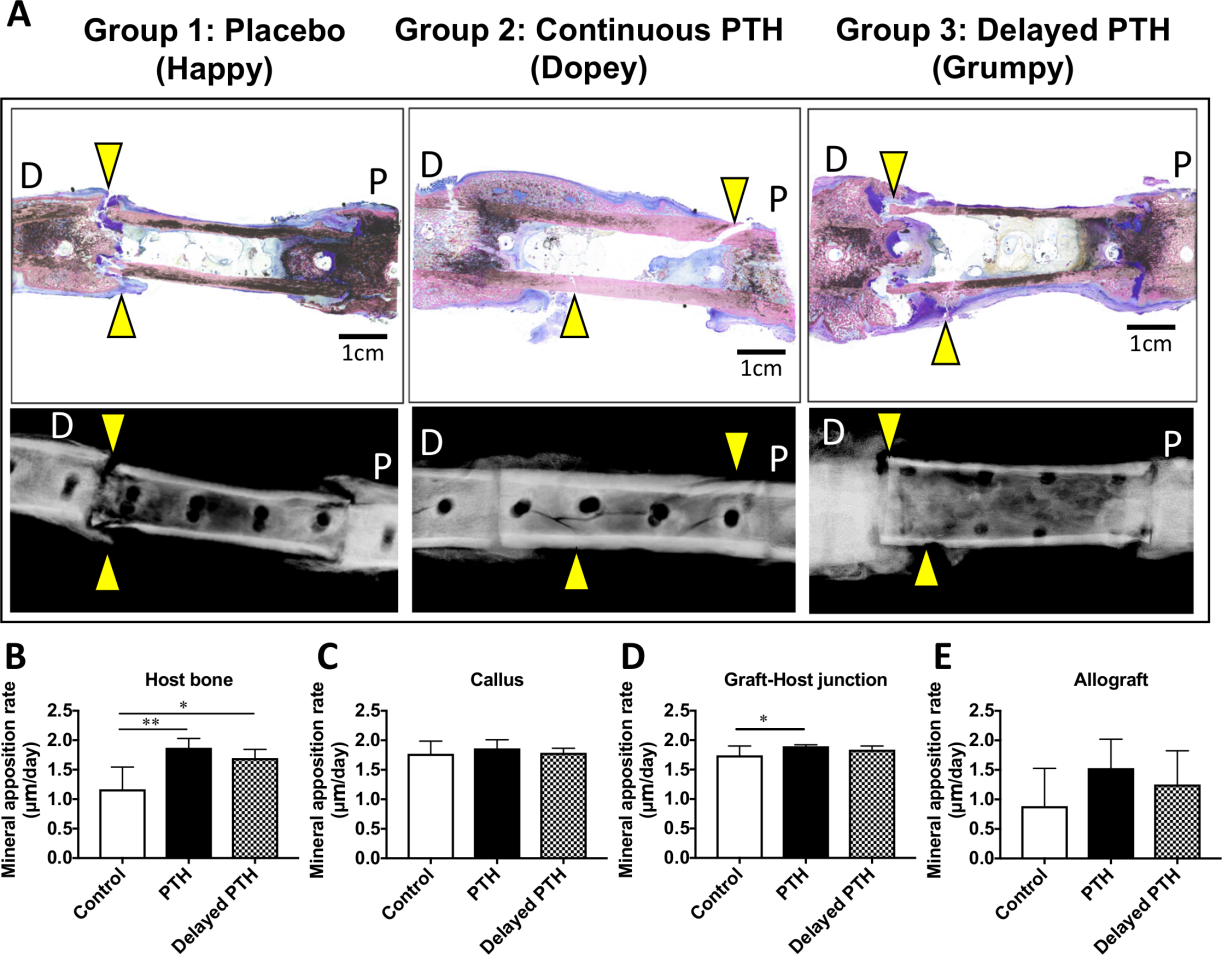
Continuous rPTH_1-34_ treatment increases the mineral apposition rate (MAR) in fracture callus at the graft-host junction. Following destructive torsion testing, the fractured allografted femurs were processed for undecalcified histology and dynamic histomorphometry as described in Materials and Methods. (A) Sanderson’s Rapid Bone Stain histology and X-rays of the representative fractured allografted femurs in each Group are shown to illustrate the amount of callus and fracture sites (yellow arrowheads) generated by the biomechanical test. Note the allograft of Happy failed at the distal graft-host junction, which is indicative of early healing or non-union. In contrast, grafted femurs from continues rPTH_1-34_ treated dog (Dopey) failed within an oblique fracture through the allograft. Delayed rPTH_1-34_ treated dog (Grumpy) failed with a fracture through the allograft and at the proximal junction. D: distal, P: proximal. MAR was quantified as described in S1 Fig, and the data for the four sub-regions: (B) Host bone; (C) Callus; (D) Graft-Host junction; and (E) Allograft, are presented as the mean +/- SD. *: p < 0.05, **: p < 0.01, by one-way ANOVA with Turkey post hoc test.

Representative Sanderson’s Rapid Bone Stain histology and plain radiographs of the fractured allografted femurs revealed that placebo treated dogs had good cortical continuity, but a modest level of callus formation in both the distal and proximal host-allograft junctions, and negligible new bone formation on the allograft (Fig 5A). This was consistent with the CB-CT data, and biomechanical failure at the graft-host junction, which is indicative of early healing or non-union. In contrast, continuous rPTH_1-34_ treated dogs displayed a remarkable amount of mature/remodeled callus that spanned the proximal and distal graft-host junctions, which produced oblique fractures through the allograft indicative of mature unions. Grafted femurs from delayed rPTH_1-34_ treated dogs contained a large unremodeled callus over the host bone that crept onto the allograft, and displayed fractures around the junction. Quantification of MAR revealed the predicted increase in host bone formation in rPTH_1-34_ treated dogs (Fig 5B). Interestingly, continuous rPTH_1-34_ treatment also increased the MAR at the Graft-Host junction versus placebo (Fig 5D). However, no differences in MAR on callus or allograft bone were observed among three groups.

## Discussion

Although complications and high failure rates of limb salvage surgery for critical defects are well known [1, 2], and have stimulated extensive investigations of biologic, gene and stem cell adjuvants [29–32], no approved therapies have emerged for many reasons including safety, cost-effectiveness, feasibility and regulatory issues. As these factors are also true for most clinical problems, repurposing FDA-approved drugs for new indications has become a focus [33]. Based on this drug development paradigm, the proven anabolic effects of rPTH_1-34_ (teriparatide) as a therapy for osteoporosis [5], and the absence of teriparatide-associated neoplasms in the FDA-mandated 15-year post-marketing Osteosarcoma Surveillance Study [34], rPTH adjuvant therapy for bone healing has been investigated in patients off-label and clinical trials. Unfortunately, evidence of efficacy remains anecdotal [9], warranting a definitive large animal study in an established clinically relevant model. Thus, we completed a canine study in the canine femoral allograft model [27], which was designed to answer safety questions about high-dose rPTH_1-34_ therapy, the potential of a short course therapy, and the utility and power of translational CB-CT outcome measures to quantify massive allograft healing. While our results do not support further investigation of high-dose therapy with this formulation of rPTH_1-34_, there were several remarkable findings that warrant discussion.

Our decision to evaluate a 5 μg/kg/day dosing regimen of rPTH_1-34_ was based on the lack of efficacy observed with lower doses [17], and published canine and human studies demonstrating its efficacy [18–20, 28]. Thus, we were very surprised to find that this treatment was poorly tolerated by the dogs. This was in contrast to the dogs in Group 1, which did not experience any complication, further supporting our conclusion that these adverse events were from rPTH_1-34_ treatment and not the surgery. While we document the first confirmed case of lethal rPTH_1-34_ hypersensitivity of a dog (S2 Fig), it should be noted that Daugaard et al also observed deaths of two dogs in their study with this dosing regime [22]. Although these observations do not establish causation, we interpret these remarkable findings to mean that canines may be highly sensitive to PTH-induced hypercalcemia, in contrast to other large animal models (i.e. rhesus monkeys that tolerate 25 μg/kg/day PTH(1–84) for 16 months [35]), and further high dose studies with this formulation of rPTH in dogs should be pursued with caution. Moreover, this toxicity highlights the need for a modified rPTH, such as recently FDA-approved abaloparatide, which has different PTH receptor bind and downstream signaling [36], decreased catabolic activity, and potentially lower incidence and severity of hypercalcemia than that seen with teriparatide [37].

In terms of efficacy, our findings that continuous rPTH_1-34_ induces robust callus formation and accelerated cortical healing at the graft-host junctions in this clinically relevant massive allograft model mirrored our prior results in the murine femoral allograft model [15]. Mechanistically, rPTH_1-34_ accelerates angiogenesis, intramembranous bone formation and endochondral ossification in the early phases of fracture healing, and then it promotes remodeling of the new bone into lamellar bone with structural integrity. However, while only 2-weeks of delayed rPTH_1-34_ was sufficient to observe maximal femoral allograft healing in mice [17], a similar treatment in our canine model had some significant effects, but was not equivalent to 8-weeks of continuous rPTH_1-34_ treatment. Most notably, delayed rPTH_1-34_ treatment had minimal callus remodeling, no increase in bone formation at the graft-host junction, and decreased cortical unions at 8-weeks compared to the continuous treatment group. This finding also highlights the importance of large animal studies to identify efficacy in small animal models, such as healing of the 4-5mm allograft used in mice that does not translate to clinically relevant critical defect healing.

Another important finding from our study comes from a comparison of the radiographic outcome measures of biomechanical healing. Based on our murine studies that showed the superiority of the 3D Union Ratio algorithm to predict biomechanical allograft healing over other CT based outcomes [24, 25], and the demonstration of its translational potential to quantify fracture healing in patients [8, 25], we hypothesized that the Union Ratio is a more accurate biomarker of biomechanical healing versus the established 2D semiquantitative scoring systems that merely evaluate the presence of callus and fracture lines subjectively. However, at the early 8-week time point, we found that the most powerful radiographic parameter of biomechanical healing is the fracture line, as we routinely found that energy to failure was best predicted by the number of bridged cortices. Interestingly, RUST score yielded better ICC (0.83) versus RUSH (0.73), and RUST also had a greater correlation with the biomechanical parameters. Thus, although the volumetric analysis allows for a wholistic appreciation of massive allograft healing from the 3D renderings, which produced convincing evidence that bridging callus across a 5cm intercalary allograft can be induced by rPTH_1-34_ in only 8-weeks (Fig 2), we do not find this approach to be cost-effective for assessment of early critical defect healing.

In addition to our negative findings, there are several noteworthy limitations of our study. First, 8-weeks is too short a recovery period to assess significant biomechanical healing. This was known during the planning phase of this study, which included a second 3 Group arm in which biomechanical healing was to be assessed at 24-weeks. However, based on the incidence and severity of the hypercalcemia observed in the rPTH_1-34_ treated dogs out to 8-weeks, the longer study was canceled due to animal welfare concerns. Second, it is possible that some significant effects of rPTH therapy were missed due to multiple saline transfusions that were administered to treat hypercalcemia, which could have altered rPTH_1-34_ pharmacokinetics. Third, while the proposed rPTH_1-34_ adjuvant therapy for massive allografting might be appropriate in trauma patients, it is important to note that teriparatide is contraindicated for use in patients with a previous history of cancer.

In conclusion, we find that continuous rPTH_1-34_ treatment significantly increases callus formation at 4 weeks, and has the potential to induce bridging callus across a 5cm intercalary allograft at 8 weeks, which has never been observed in untreated or placebo controls. 2D radiographic scoring of four cortices healing (RUST) is a reliable cost-effective outcome of early intercalary allograft healing. Based on the demonstrated feasibility and efficacy, development of a rPTH formulation with reduced hypercalcemic side effects is warranted towards the development of a safe high dose therapy for critical defects.

## Supporting information

S1 Fig Fluorescent imaging of undecalcified double-labeled bone sections and automated histomorphometry to quantify the mineral apposition rates (MAR) on host, callus, graft-host junction and allograft bone. (A) Photograph of the unstained histology section of Dopey’s allografted femur highlighting the 8 regions of interest (ROI) that were analyzed for dynamic histomorphometry (H1, H2 = proximal and distal host bone; C1, C2 = proximal and distal callus bone; J1, J2 = proximal and distal junctions; A1, A2 = allograft cortices). (B) 10x fluorescent image of the calcein green and alizarin red double labeling is shown to illustrate the primary data used for automated histomorphometry. To segment alizarin and calcein labels and quantify the mineral apposition rate (MAR = distance between adjacent labeling lines representing bone growth during the 7 day interval between alizarin and calcein injections), a fully automated algorithm was developed. Briefly, a series of spectral filters were applied to enhance and normalize alizarin and calcein fluorescence in each image. Alizarin and calcein channels were subsequently separated and processed using boundary smoothing, hole filling, and area/length-based filters. The resulting alizarin and calcein masks were “skeletonized” (with pruning), creating 1-pixel thick lines along the medial axis of each labeling line (C). A k-neighbor search algorithm (with the maximum distance set to 100 μm) was then applied to match each alizarin skeletal pixel to the closest proximity calcein skeletal pixel. This process produced a bone growth mask representing distances between labeling lines. To quantify growth (i.e., the width or thickness of each growth region), a Euclidean distance filter was applied to the bone growth mask and then subsequently “multiplied” by the skeleton of the bone growth mask, such that the intensity along the resulting 1-pixel lines represented the growth distance between the medial axes of the adjacent alizarin and calcein labeling lines. Bone growth regions were pseudo-colored based on their thickness values and superimposed upon a grayscale representation of the original input image (D) with accompanying thickness heat map. Measurements of the intensity of these skeletonized lines (both on a pixel-by-pixel basis and by distinct connected regions), raw bone growth distances (accompanied by the corresponding length of each distinct region), and average/standard deviations of mineral apposition rates (“MAR”; bone growth distance/interval in mm/day) were quantified. Additionally, the overall length and area of calcein and alizarin were also quantified.
(TIF)

S2 Fig Case report of a canine death associated with complications from hypercalcemia following 3 doses of rPTH (5 μg/kg). La, a dog in Group 3, Cohort 2, underwent femoral allograft surgery on September 11, 2013, and had a good recovery. La commenced the delay rPTH therapy (5 μg/kg/day s.c.) on day 14 post-op as scheduled. The day after the 3^rd^ rPTH injection, La had lethargy and diarrhea, and rPTH treatment was discontinued. Hypercalcemia peaked at a serum ionized calcium level of 1.69 mmol/L on day 18 post-op, which was treated with fluids, and attitude improved. However, general health continued to deteriorate with poor appetite, oliguria, tachycardia and low blood pressure, culminating with shock/lateral recumbency and death on day 19 post-op. Immediate necropsy included histopathology evaluation of the colon, heart, kidney, liver, lung, lymph node, pancreas, small intestine, spleen and stomach. In summary, the animal had metastatic calcification in the kidneys, heart, vasculature and stomach, which was found to be consistent with excessive exogenous PTH administration. The hypercalcemia in this animal may have caused significant arrhythmias and subsequent myocardial failure and shock. Acute tubular necrosis in the kidney and necrohemorrhagic gastritis were also observed, and may have been the result of the calcification and/or shock. Acute alveolar injury in the lungs was observed and most likely the sequelae to shock. The following were tissue specific findings.
**Heart:** Multifocally, there was moderate mineralization of myofibers and intimal lining of the vasculature, and rare macrophages and neutrophils associated with mineralized degenerating myofibers, as illustrated in the 10x and 20x micrographs of H&E stained histology.
**Kidney:** Multifocally, there was moderate renal tubular, glomerular and collecting duct mineralization. There was also multifocal attenuation of renal tubular epithelium and tubular epithelial sloughing (acute tubular necrosis) with scattered tubular luminal and glomerular (Bowman's space) protein accumulation. In a few foci there were mild interstitial lymphoplasmacytic infiltrates.
**Liver:** There was marked congestion of the sinusoids. Focally, there was a small area of necrosis.
**Lung**: Diffusely, alveoli were expanded and congested, and contained abundant fibrin and edema with neutrophils and alveolar macrophage infiltrates.
**Small Intestine**: There were mild mucosal infiltrates with lymphocytes, plasma cells, neutrophils and eosinophils.
**Stomach**: Diffusely, in the section of fundic stomach reviewed, there was necrosis of gastric pits with marked hemorrhage. Glandular epithelial cells were sloughing and necrotic, and there were neutrophils infiltrating the mucosa. There was diffuse mineralization of basement membrane of glands and multifocal mineralization of the muscularis. No lesions were seen in the pyloric section.
**Lymph Node, Colon, Pancreas, Spleen**: No significant lesions were noted.
(TIF)

S3 Fig Intermittent PTH treatment has no effect on CB-CT derived Union Ratio at 8-weeks of allograft healing. The Union Ratio was calculated from the 8-week post-op CB-CT scans as described in Materials and Methods, and the data are presented as mean ± SD. No significant differences were observed.
(TIF)
