# Supplementary figures and images for: High dose teriparatide (rPTH1-34) therapy increases callus volume and enhances radiographic healing at 8-weeks in a massive canine femoral allograft model
Source: PLoS One. 2017 Oct 11;12(10):e0185446. doi: 10.1371/journal.pone.0185446 (PMC5636088; doi:10.1371/journal.pone.0185446)

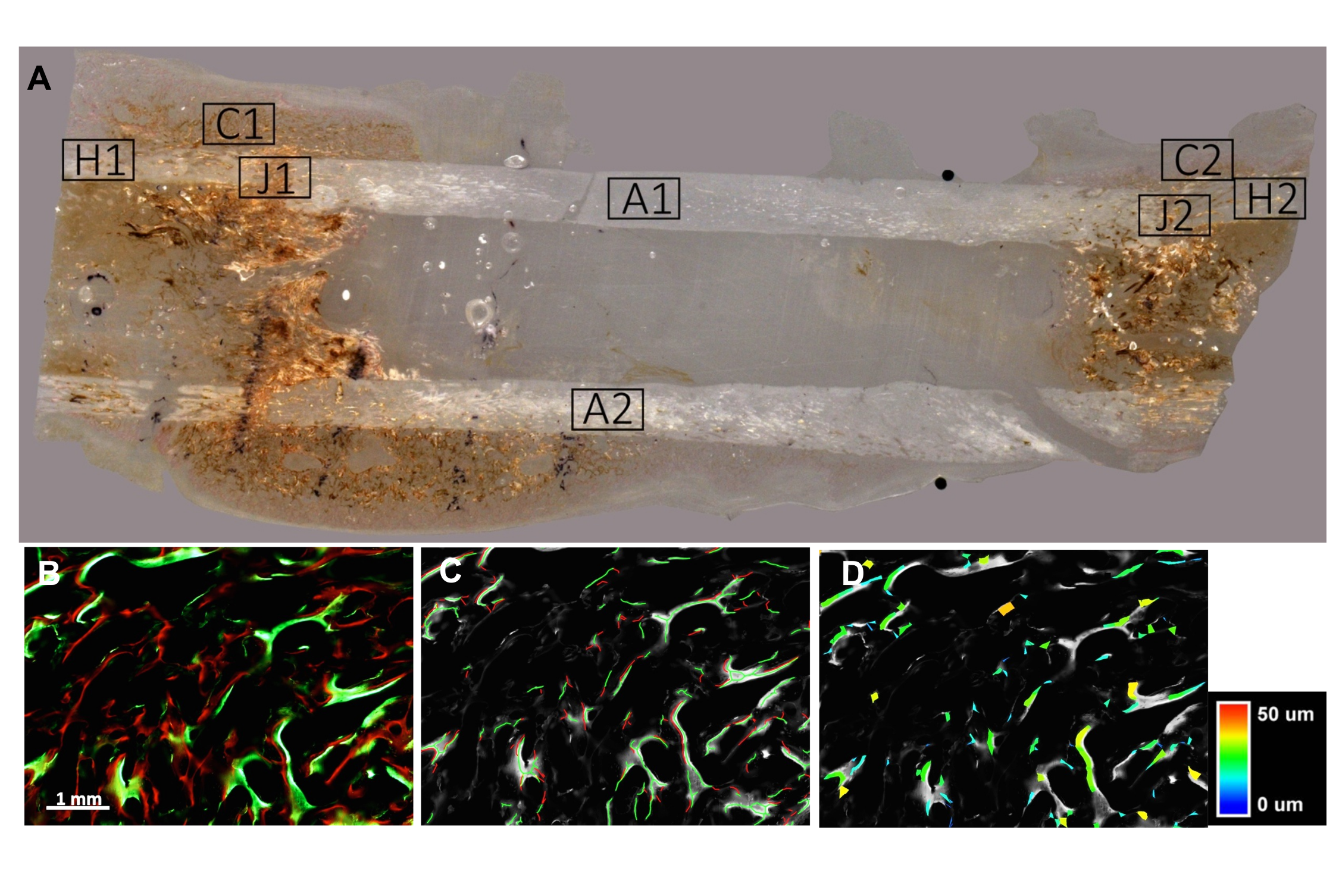

Supplement: S1 Fig — (A) Photograph of the unstained histology section of Dopey’s allografted femur highlighting the 8 regions of interest (ROI) that were analyzed for dynamic histomorphometry (H1, H2 = proximal and distal host bone; C1, C2 = proximal and distal callus bone; J1, J2 = proximal and distal junctions; A1, A2 = allograft cortices). (B) 10x fluorescent image of the calcein green and alizarin red double labeling is shown to illustrate the primary data used for automated histomorphometry. To segment alizarin and calcein labels and quantify the mineral apposition rate (MAR = distance between adjacent labeling lines representing bone growth during the 7 day interval between alizarin and calcein injections), a fully automated algorithm was developed. Briefly, a series of spectral filters were applied to enhance and normalize alizarin and calcein fluorescence in each image. Alizarin and calcein channels were subsequently separated and processed using boundary smoothing, hole filling, and area/length-based filters. The resulting alizarin and calcein masks were “skeletonized” (with pruning), creating 1-pixel thick lines along the medial axis of each labeling line (C). A k-neighbor search algorithm (with the maximum distance set to 100 μm) was then applied to match each alizarin skeletal pixel to the closest proximity calcein skeletal pixel. This process produced a bone growth mask representing distances between labeling lines. To quantify growth (i.e., the width or thickness of each growth region), a Euclidean distance filter was applied to the bone growth mask and then subsequently “multiplied” by the skeleton of the bone growth mask, such that the intensity along the resulting 1-pixel lines represented the growth distance between the medial axes of the adjacent alizarin and calcein labeling lines. Bone growth regions were pseudo-colored based on their thickness values and superimposed upon a grayscale representation of the original input image (D) with accompanying thickness heat m [file pone.0185446.s001.tif]

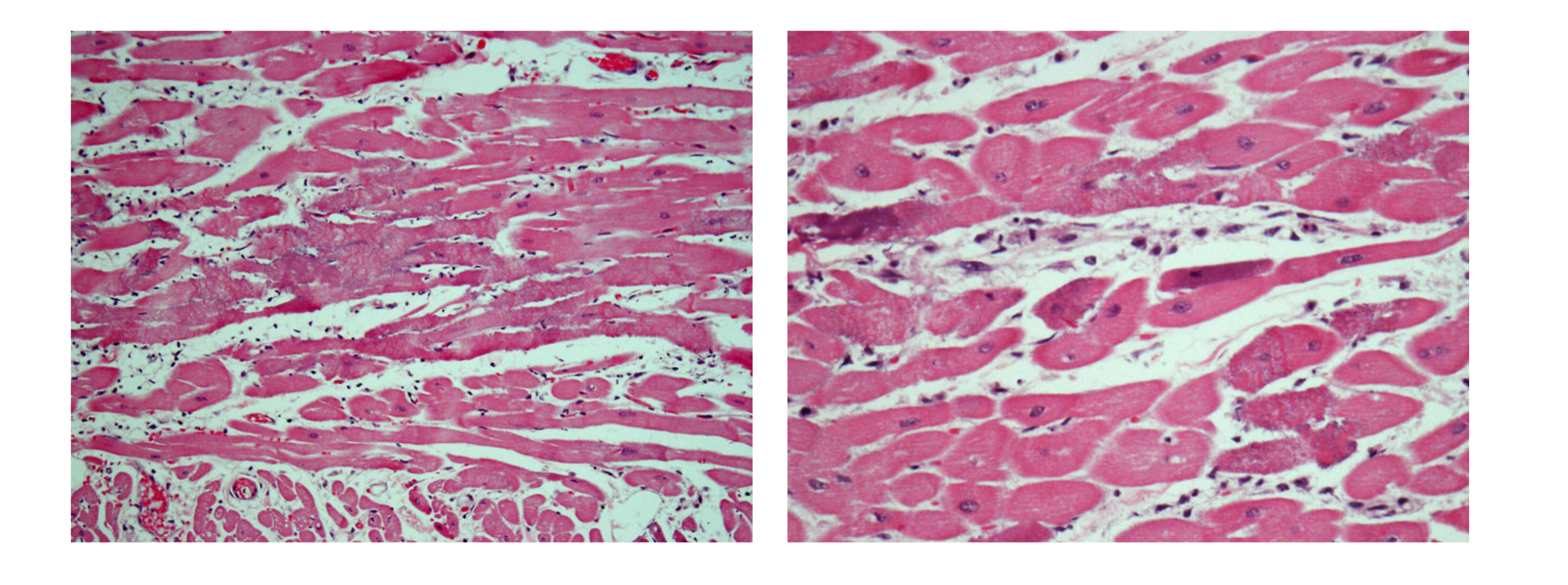

Supplement: S2 Fig — La, a dog in Group 3, Cohort 2, underwent femoral allograft surgery on September 11, 2013, and had a good recovery. La commenced the delay rPTH therapy (5 μg/kg/day s.c.) on day 14 post-op as scheduled. The day after the 3rd rPTH injection, La had lethargy and diarrhea, and rPTH treatment was discontinued. Hypercalcemia peaked at a serum ionized calcium level of 1.69 mmol/L on day 18 post-op, which was treated with fluids, and attitude improved. However, general health continued to deteriorate with poor appetite, oliguria, tachycardia and low blood pressure, culminating with shock/lateral recumbency and death on day 19 post-op. Immediate necropsy included histopathology evaluation of the colon, heart, kidney, liver, lung, lymph node, pancreas, small intestine, spleen and stomach. In summary, the animal had metastatic calcification in the kidneys, heart, vasculature and stomach, which was found to be consistent with excessive exogenous PTH administration. The hypercalcemia in this animal may have caused significant arrhythmias and subsequent myocardial failure and shock. Acute tubular necrosis in the kidney and necrohemorrhagic gastritis were also observed, and may have been the result of the calcification and/or shock. Acute alveolar injury in the lungs was observed and most likely the sequelae to shock. The following were tissue specific findings. Heart: Multifocally, there was moderate mineralization of myofibers and intimal lining of the vasculature, and rare macrophages and neutrophils associated with mineralized degenerating myofibers, as illustrated in the 10x and 20x micrographs of H&E stained histology. Kidney: Multifocally, there was moderate renal tubular, glomerular and collecting duct mineralization. There was also multifocal attenuation of renal tubular epithelium and tubular epithelial sloughing (acute tubular necrosis) with scattered tubular luminal and glomerular (Bowman's space) protein accumulation. In a few foci there were mild interstitial lympho [file pone.0185446.s002.tif]

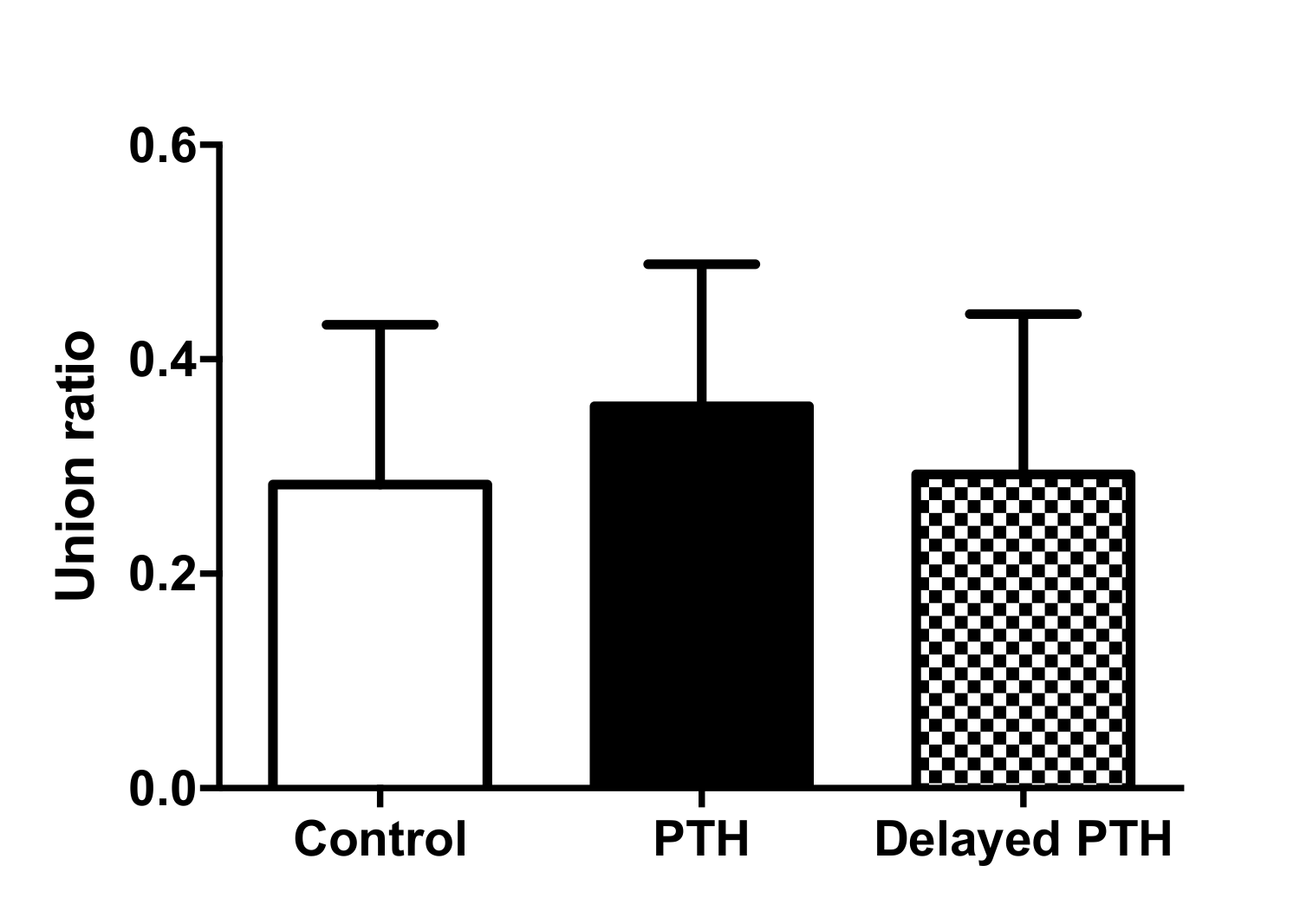

Supplement: S3 Fig — The Union Ratio was calculated from the 8-week post-op CB-CT scans as described in Materials and Methods, and the data are presented as mean ± SD. No significant differences were observed. (TIF) [file pone.0185446.s003.tif]
